# Neuroendocrine Control of Macrophage Development and Function

**DOI:** 10.3389/fimmu.2018.01440

**Published:** 2018-06-25

**Authors:** Arnon Dias Jurberg, Vinícius Cotta-de-Almeida, Jairo Ramos Temerozo, Wilson Savino, Dumith Chequer Bou-Habib, Ingo Riederer

**Affiliations:** ^1^Laboratory on Thymus Research, Oswaldo Cruz Institute/Oswaldo Cruz Foundation, Rio de Janeiro, Brazil; ^2^Brazilian National Institute of Science and Technology on Neuroimmunomodulation (INCT-NIM), Rio de Janeiro, Brazil

**Keywords:** macrophages, monocytes, neuroendocrine system, hormones, neurotransmitters, stress, glucocorticoids

## Abstract

Macrophages carry out numerous physiological activities that are essential for both systemic and local homeostasis, as well as innate and adaptive immune responses. Their biology is intricately regulated by hormones, neuropeptides, and neurotransmitters, establishing distinct neuroendocrine axes. The control is pleiotropic, including maturation of bone marrow-derived myeloid precursors, cell differentiation into functional subpopulations, cytotoxic activity, phagocytosis, production of inflammatory mediators, antigen presentation, and activation of effector lymphocytes. Additionally, neuroendocrine components modulate macrophage ability to influence tumor growth and to prevent the spreading of infective agents. Interestingly, macrophage-derived factors enhance glucocorticoid production through the stimulation of the hypothalamic–pituitary–adrenal axis. These bidirectional effects highlight a tightly controlled balance between neuroendocrine stimuli and macrophage function in the development of innate and adaptive immune responses. Herein, we discuss how components of neuroendocrine axes impact on macrophage development and function and may ultimately influence inflammation, tissue repair, infection, or cancer progression. The knowledge of the crosstalk between macrophages and endocrine or brain-derived components may contribute to improve and create new approaches with clinical relevance in homeostatic or pathological conditions.

## Introduction

Integration of body functions relies on distinct mechanisms encompassing various organs and systems. As a consequence, perturbations in the environment or in tissue homeostasis usually produce quick and effective responses that may result in significant local and systemic impacts, for example, due to a sustained communication between the central nervous system (CNS) and peripheral organs through the concerted activities of cell type-specific chemical messengers. Major neuroendocrine axes comprise the hypothalamus and the pituitary in the brain signaling to adrenal glands [hypothalamus–pituitary–adrenal (HPA) glands or HPA axis], thyroid [hypothalamus–pituitary–thyroid (HPT) axis], or gonads [hypothalamus–pituitary–gonad (HPG) axis]. They exert regulatory effects on the immune system, and any imbalance disrupting such neuroimmunoendocrine communication may result in pathological conditions (1–3).

Among the cells of the immune system involved in neuroendocrine interactions, macrophages play a central role in the activation and modulation of both innate and adaptive immune responses. Interestingly, an intricate bidirectional macrophage-neuroendocrine system crosstalk is currently being explored to understand homeostasis and diseases. Here, we review how macrophages bridge the immune, endocrine, and nervous systems, how hormones and neurotransmitters may influence their physiology and function and to what extent such circuitry may be placed as potential therapeutic targets in various diseases. We do not extend our analysis on how macrophage-derived mediators affect brain activity and behavior, since recent comprehensive reviews about this issue have been published (4–6).

## Macrophages and Neuroendocrine Components

Macrophages are multifunctional leukocytes that recognize and remove invading pathogens, toxins, cellular debris and apoptotic cells in healthy or inflamed tissues. They are tissue-resident cells that have settled during embryogenesis or monocyte-derived cells that migrated from the blood circulation and reached different organs (7). Depending on the organ they populate, macrophages receive different designations, which are supported by specific differentiation programs, cell morphologies, and specialized functions (8, 9). To name a few, they are known as microglia in the brain, alveolar macrophages in the lungs, Kupffer cells in the liver, osteoclasts in bones, and chondroclasts in cartilages. More specifically, macrophages are further classified into distinct subpopulations based on their functional properties, which may comprise non-activated circulating monocytes, pro-inflammatory or anti-inflammatory macrophages, among others. Indeed, strong plasticity in their differentiation and the notion that these subsets may be interchangeable have led to the proposition of a “spectrum wheel” system (10–12). In this regard, additional cellular subsets can be defined by a combination of characteristics, as it will be discussed below.

To perform their roles, macrophages rely on a wide range of specific surface and intracellular receptors. These sensors are able to recognize microbial components, defined as pathogen-associated molecular patterns, and danger molecules released after cell and tissue lesions, defined as damage-associated molecular patterns. Macrophages activated through these receptors produce potent pro-inflammatory cytokines, such as TNF-α, IL-1β, IL-6, and IL-12, together with chemokines and toxic-free radicals (13–15). Furthermore, other receptors play critical roles on macrophage function. For example, scavenger receptors (SRs) bind a diverse range of ligands from bacteria to native proteins, allowing them to regulate both cell adhesion and removal of noxious agents by phagocytosis (16). A class of SRs includes the mannose receptor (CD206), which is a marker of M2 macrophages (see below). In fact, when CD206-positive macrophages were eliminated from the lungs of a murine transgenic model of toxemia by the administration of diphtheria toxin, mice developed an exacerbated lung inflammation upon endotoxin challenge (17). Death of neighboring cells by apoptosis is initially perceived by macrophages through specific receptors that recognize phosphatidylserine exposure in the lipid bilayer membrane of dying cells. Macrophages are also able to detect complement molecules through cognate receptors and antibodies through Fc receptors. These molecules opsonize pathogens and abnormal cells, thus stimulating their phagocytosis.

**Figure 1:**
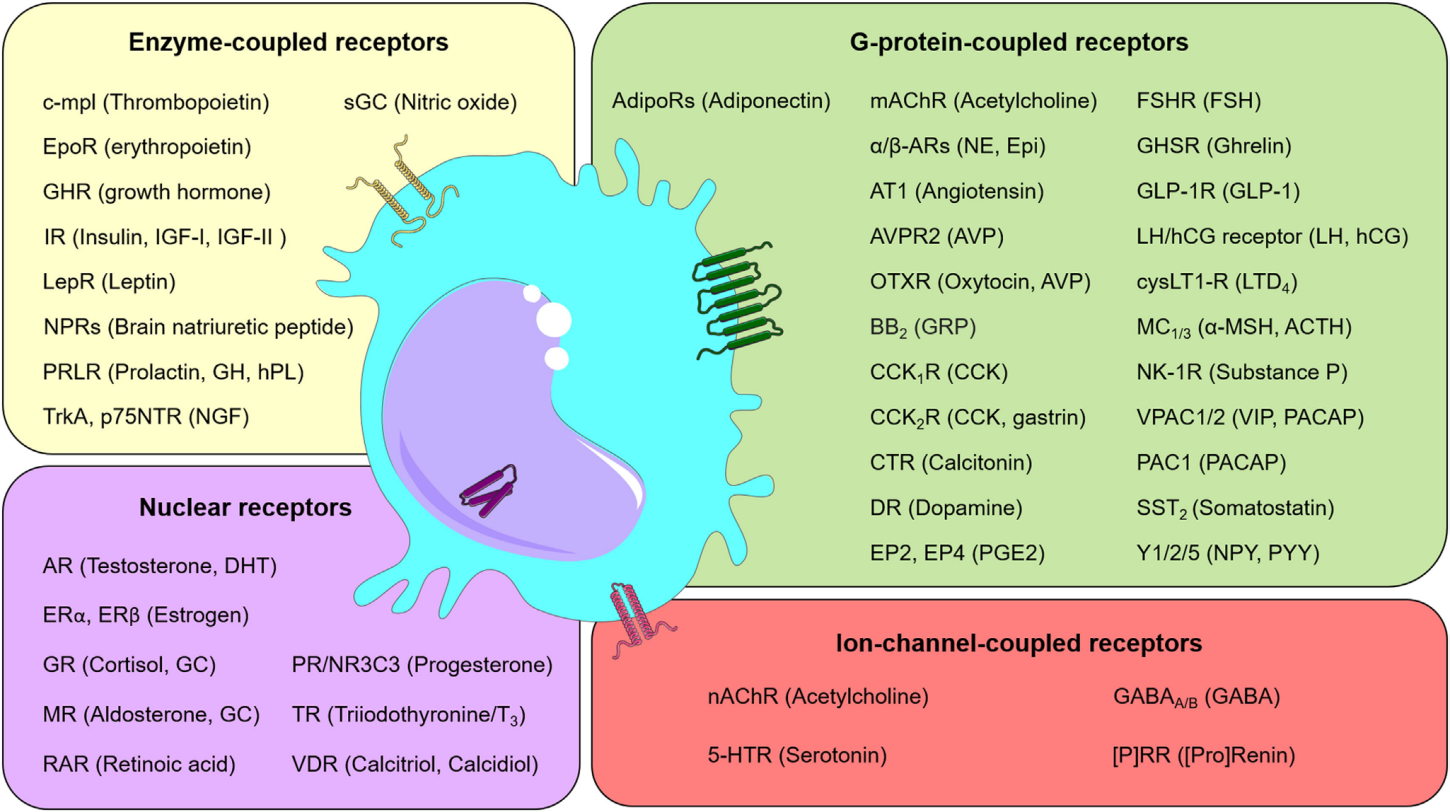
Neuroendocrine communication on macrophages. Schematic representation listing selected receptors (and their ligands) found in macrophages. Receptors were grouped into classes, as indicated. Abbreviations: (P)RR, (pro)renin receptor; 5-HTR, serotonin receptor; ACTH, adrenocorticotropic hormone; AdipoRs, adiponectin receptors; AR, androgen receptor; AT1, angiotensin II receptor type 1; AVP, arginine vasopressin or antidiuretic hormone; AVPR2, arginine vasopressin receptor 2; BB2, bombesin receptor; CCK, cholecystokinin; CCK_1/2_R, cholecystokinin receptor 1/2, respectively; c-mpl, myeloproliferative leukemia protein; CO, carbon monoxide; CTR, calcitonin receptor; cysLT1-R, cysteinyl leukotriene receptor 1; DHT, dihydrotestosterone; DR, dopamine receptor; EP2, prostaglandin E2 receptor 2; EP4, prostaglandin E2 receptor 4; Epi, epinephrine; EpoR, erythropoietin receptor; ER, estrogen receptor; FSH, follicle-stimulating hormone; FSHR, follicle-stimulating hormone receptor; GABA, gamma-aminobutyric acid; GABA_A/B_, GABA_A_-receptor and GABA_B_-receptor, respectively; GC, glucocorticoids; GH, growth hormone; GHR, growth hormone receptor; GHSR, growth hormone secretagogue receptor (also known as ghrelin receptor); GLP-1, glucagon-like peptide-1; GLP-1R, Glucagon-like peptide-1 receptor; GR, glucocorticoid receptor; GRP, gastrin-releasing peptide; hCG, human chorionic gonadotropin; hPL, human placental lactogen; IGF, insulin-like growth factor; IR, insulin receptor; LepR, leptin receptor; LH, luteinizing hormone; LTD_4_, leukotriene D_4_; mAChR, muscarinic acetylcholine receptor; MC_1/3_, melanocortin 1/3 receptor, respectively; MR, mineralocorticoid receptor; nAChR, nicotinic acetylcholine receptor; NE, norepinephrine; NGF, nerve growth factor; NK-1R, neurokinin 1 receptor; NPRs, natriuretic peptide receptors; NPY, neuropeptide Y; NR3C3, nuclear receptor subfamily 3, group C, member 3; OTXR, oxytocin receptor; p75NTR, neurotrophin receptor p75; PAC1, pituitary adenylate cyclase-activating polypeptide type I receptor; PACAP, pituitary adenylate cyclase-activating peptide; PGE2, prostaglandin 2; PR, progesterone receptor; PRLR, prolactin receptor; PYY, Peptide YY; RAR, retinoic acid receptor; sGC, soluble guanylyl cyclase; Soluble guanylyl cyclase (GC-1); SST_2_, somatostatin receptor type 2; TR, thyroid hormone receptor; TrkA, transmembrane tyrosine kinase; VDR, vitamin D receptor; VIP, vasoactive intestinal peptide; VPAC1/2, vasoactive intestinal peptide receptor 1/2, respectively; Y1/2/5, neuropeptide Y receptor type 1/2/5, respectively; α/β-ARs, α/β-adrenergic receptors; α-MSH, melanocyte-stimulating hormone.

Analyses of a large body of evidence revealed that macrophages could respond to a wide variety of neuroendocrine factors [e.g., Ref. (18)]. In particular, a second-level evaluation of published data sets available through the Immunological Genome Project (ImmGen) Consortium (19) shows that monocytes and macrophages express not only numerous hormone and neurotransmitter receptors (Figure 1) but also a number of the corresponding cognate ligands. More specifically, using neuroendocrine-related gene expression profiles, it is possible to cluster myeloid cells by cell type and body locations (Figure S1 in Supplementary Material). Accordingly, the genes for angiotensin I converting enzyme (*Ace*), 15-hydroxyprostaglandin dehydrogenase (*Hpgd*), and EGF-like module containing, mucin-like, hormone receptor-like sequence 4 (*Emr4*/*Adgre4*) are highly expressed in different populations of circulating monocytes. Contrasting with this transcriptomic observation, Stacey et al. (20) have identified higher expression levels of *Emr4/Adgre4* predominantly in resident macrophages. In a second hand, genes such as *Ltc4s, Ptgs1, Igf1, Ophn1, Hpgds, Gatm, Pgcp*, and *Cysltr1* were highly expressed in most macrophages and some populations of dendritic cells, but slightly expressed in monocytes. Together, the presence of neuroendocrine components in monocytes and macrophages provide the grounds for the notion that macrophage-neuroendocrine crosstalk influences the overall homeostasis and immunity of an individual.

In the sections below, we will discuss in more detail how hormones, nervous-derived cytokines, and neurotransmitters regulate different aspects of macrophage biology related to the preservation of internal homeostasis.

## Neurotransmitters and Hormones Regulate Macrophage Function

The vast number of neuroendocrine factors places a significant challenge for the quest to unravel brain-immune communication. Nevertheless, it may also unveil numerous possibilities for clinical intervention. The early isolation of specific hormones and the availability of recombinant proteins, as well as gene editing technologies, have allowed the study of various molecules of interest in macrophage physiology. The first studies showing that macrophages were able to respond to neurotransmitters date back to mid-past century with the finding that phagocytosis was stimulated by histamine (21). This small monoamine messenger is produced by some immune cells (e.g., mast cells and basophils) and by neurons of the tuberomammillary nucleus of the hypothalamus (22, 23). The biological significance of histamine to macrophage function was later demonstrated in distinct models of intracellular infection (24–26) and paved the way for the investigation of other neurotransmitters endowed with similar properties to modulate macrophage physiology.

The discovery that macrophages also respond to hormonal stimuli came shortly after. Then, a large body of publications showed that hormones can broadly modulate both the immune system and inflammatory responses [e.g., Ref. (27–31)]. Among them, early clinical observations and experimental investigations provided evidence that the formation of the so-called granulation tissue was impaired upon treatment with cortisone or adrenocorticotropic hormone (ACTH) (29, 32). The absence of this delimited transitory regenerative response full of macrophages and new blood vessels raised the assumption that corticosteroids could weaken macrophage function, a hypothesis that was later undermined by the finding that corticosteroids were shown to promote macrophage migration in culture (33, 34). Indeed, the effects of corticosteroids on the formation of granulation tissue seem to rely on suppressing blood vessel formation through the inhibition of platelet-derived growth factor-dependent expression of vascular endothelial growth factor (35). Since those discoveries, reports concerning neuroendocrine modulation of the immune system have become available, and many comprehensive reviews have been published (36, 37). Therefore, herein, we will focus on the influence of neuroendocrine messengers on macrophage physiology by dividing the distinct stages of their lifespan into the following sub-sections.

### Monocyte/Macrophage Maturation May Be Driven by Neuroendocrine Components

Although macrophage origin remains a matter of intense debate, they seem to arise from at least two distinct locations. Early in life, *Myb*-independent yolk sac-derived erythro-myeloid Csf1r-positive progenitors emerge from blood islands, colonize the developing liver at early- to mid-gestation, and subsequently reach other organs, such as lungs, epidermis, and brain (9, 38–43). These macrophages can persist and proliferate in either healthy young adults or upon tissue insult (40, 44–46). Later, new macrophages and dendritic cells differentiate from bone marrow-derived circulating monocytes upon reaching a target tissue damaged by inflammatory reactions or pathogens.

**Figure 2:**
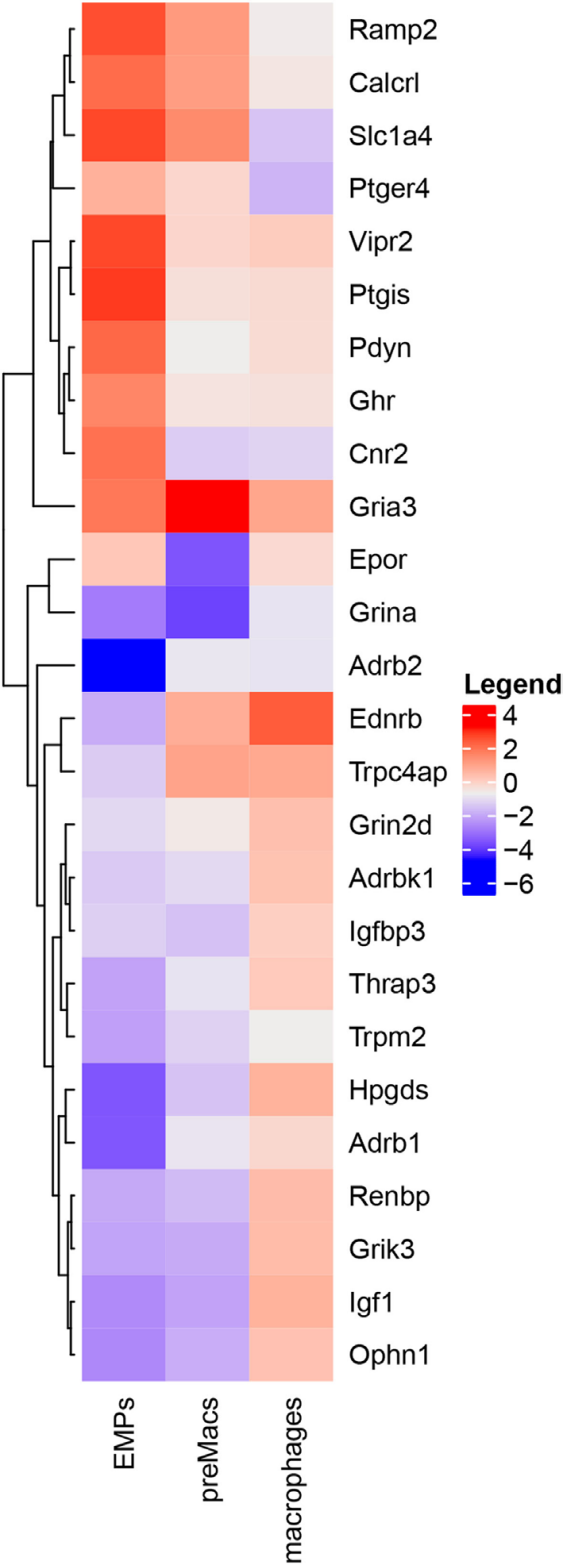
Neuroendocrine-associated genes are differentially expressed during macrophage differentiation. Heatmap of RNA-Seq profile filtered by keywords from Mass et al. (9) depicting hierarchically clustered relative gene expression (log2) in erythro-myeloid progenitors, pre-macrophages (preMacs), and macrophages. Levels of expression are represented by colors in which red, white, and blue indicate high, intermediate, and low intensities, respectively.

The influence of neuroendocrine messengers on early macrophage differentiation is largely underappreciated, but a closer look at the data published by Mass et al. (9) reveals that many neuroendocrine-related genes are differentially expressed during macrophage stepwise maturation (Figure 2). Some of them, like calcitonin-related genes (*Calcrl* and *Ramp2*), prostaglandin-associated genes (*Ptgis* and *Ptger4*), and both *Vipr2* and *Ghr* are highly expressed in erythro-myeloid progenitors (EMPs), but gradually decline as cells differentiate into macrophages. The glutamate receptor gene *Gria3* exhibits high expression levels in EMPs, a further increase in CD45^+^Kit^−^Lin^−^ pre-macrophages (pMacs), and a subsequent reduction in mature macrophages. In turn, the erythropoietin receptor gene (*Epor*) is detected in intermediate levels either in EMPs or macrophages, but it shows a low expression in pMacs. Other genes, such as *Adbr2* (β2-adrenergic receptor), *Ednrb* (endothelin receptor type B), and *Igf1* (insulin-like growth factor 1), exhibit lower levels in EMPs and pMacs, but higher expression in macrophages. Together, these expression profiles suggest that neuroendocrine signals modulate macrophage maturation and may affect macrophage function. Experimental investigations using selective activation or inactivation of genes of interest in conditional systems are, therefore, necessary to elucidate possible medical benefits from manipulating neuroendocrine influence on macrophage differentiation.

### Macrophage Polarization in Response to Neuroendocrine Stimuli

It is acknowledged that it is through cell polarization that proper macrophage effector responses can be achieved in target tissues. However, macrophage nomenclature may be confusing to reconcile between *in vitro*-induced phenotypes and their *in vivo* relevance. From now on, we will refer to macrophage subsets based on their functions or cellular markers, thus avoiding whenever possible the misconceptions that may arise from distinct stimulation conditions among laboratories (47).

The preference toward a given functional phenotype of macrophages is classically triggered by cytokines and specific pathogens. Formerly known as M1 or classically activated macrophages, cells involved in host defense against microbes and tumors show a pro-inflammatory phenotype induced by IL-12 and IFN-γ [M(IFNγ) macrophages]. Interestingly, they also participate in the onset of tissue repair upon an injury or trauma (47, 48). In turn, a heterogeneous population of anti-inflammatory macrophages may arise secondary to stimulation with Th2 cytokines, glucocorticoids, immune complexes, colony stimulating factor-1, or by some intracellular microorganisms, such as *Leishmania* (49, 50). Initially associated with wound healing and known as alternatively activated or M2 macrophages, these anti-inflammatory cells show increased arginase-1 activity, which generates ornithine and urea. Ornithine is subsequently converted to proline and polyamines, which are used in the biosynthesis of collagen and in cell proliferation, respectively (51, 52). By converting arginine to ornithine, arginase-1 competes with the nitric oxide (NO)-producing enzyme NO synthase characteristic of pro-inflammatory macrophages (53). Other anti-inflammatory macrophages may be produced by the incubation with IL-10 [M(IL-10)] or glucocorticoids and TGF-β [M(GC + TGFβ)], thereby polarizing them to a pro-healing phenotype with high scavenging activity also known as “deactivated, regulatory, or M2c macrophages” [reviewed by Martinez et al. (54)]. Unlike M(IFNγ), M(IL-4), or M(IL-10) macrophages, glucocorticoid-induced macrophages express higher levels of Mer tyrosine kinase (MerTK), a surface receptor involved in the phagocytosis of early apoptotic cells through the recognition of exposed phosphatidylserine (55). Despite their specificities, these phenotypes are plastic, and macrophages can switch between distinct functions both *in vivo* and *in vitro* upon a number of distinct stimuli (10–12, 56–58).

**Figure 3:**
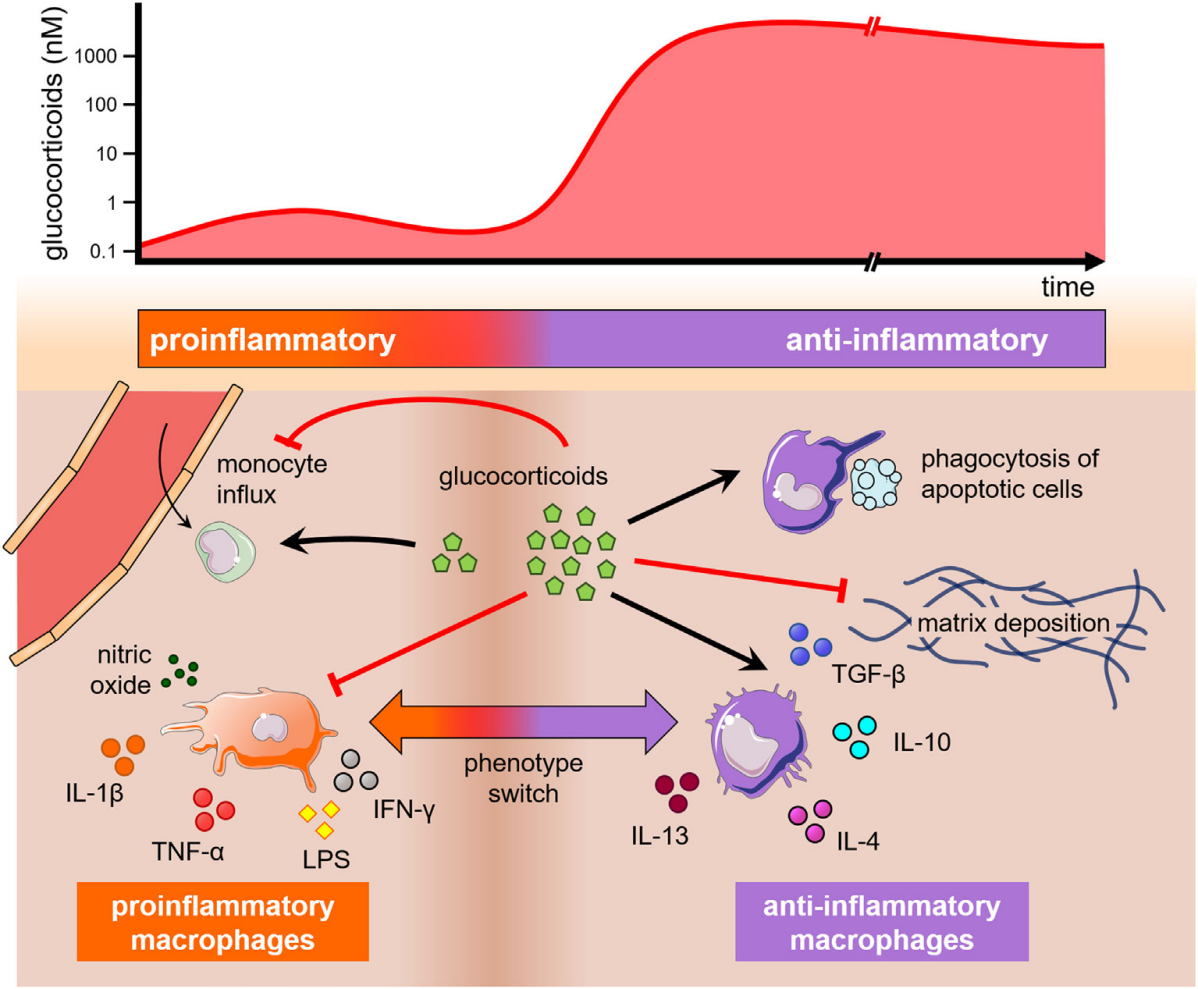
The influence of glucocorticoids on monocytes and macrophages. Glucocorticoids may play opposing effects on monocytes and macrophages depending on theirs levels and time of exposure (top). A graphical representation depicts these effects on monocyte trafficking into tissues, macrophage polarization, and phagocytosis (bottom). A short and low increase in glucocorticoid levels (left) stimulates monocyte extravasation into the injured tissue, while high and long-lasting levels of glucocorticoids (right) inhibit monocyte proliferation and extravasation, as well as drive macrophage polarization into anti-inflammatory phenotypes that may produce extracellular matrix or stimulate the engulfment of apoptotic cells. All these phenomena are also influenced by cytokines present in the milieu.

The finding that glucocorticoids influence macrophage polarization points out that neuroendocrine components may also contribute to macrophage subset decision and hence tissue regeneration. In this regard, Gratchev et al. (59) have demonstrated that M(IL-4) macrophages secrete extracellular matrix (ECM) components and remodeling enzymes, as tenascin-C and metalloproteases, respectively, whereas M(GC) macrophages exhibited undetected or reduced levels of many ECM-associated proteins. Of note, cell-specific gene inactivation of the mouse glucocorticoid receptor (GR) in the myeloid lineage impaired cardiac healing after experimental ischemic injury, due to abnormal collagen scar formation, reduced neovascularization, and persistent pro-inflammatory differentiation of macrophages. Moreover, dexamethasone can overcome the effects of IL-4 on the production of macrophage-derived ECM molecules. Unlike M(IL-4) cells, only M(IL-4 + dexa) or M(GC) macrophages were responsive to TGF-β, a major cytokine in the resolution phase (Figure 3) (60). Thereby, dexamethasone modulates the resolution phase by inhibiting the expression of NF-κB-dependent pro-inflammatory cytokines involved in the initial dominant inflammatory phase while inducing a resolutive and reparative phenotype (61–64). This limits inflammation and highlights the preponderant role of glucocorticoids on the regulation of macrophage function.

The modulatory properties of neurotrophic factors and neuropeptides are also prominent in the regulation of immune cells, including monocytes and macrophages. For instance, norepinephrine by itself is a potent inducer of alternatively activated macrophages even in the presence of LPS (65). By contrast, activation of monocytes and macrophages by LPS induces increased production of the neurotrophin nerve growth factor (NGF), which favors a pro-inflammatory phenotype through the induction of monocyte cytotoxic potential and the production of TNF-α (66, 67). Importantly, macrophage production of NGF protects them against apoptosis during inflammation or HIV-1 infection (67, 68). However, the protective survival role of NGF in HIV-1-infected macrophages may be counterbalanced by its stimulating effect on viral replication, which occurs through the downregulation of the cytidine deaminase APOBEC3G (69). Neurotrophins may actually play opposing roles in their abilities to control the growth of pathogens in macrophages, since NGF protects macrophage from infection with the protozoan *Leishmania donovani* through the increased production of hydrogen peroxide (70). Meanwhile, the widely distributed vasoactive intestinal peptide and the pituitary adenylate cyclase-activating polypeptide (PACAP) downregulate macrophage-derived production of numerous pro-inflammatory molecules, whereas inducing macrophage synthesis of the anti-inflammatory mediators IL-4 and IL-10 (71–73). Overall, neuropeptides are able to modulate macrophage-dependent activities and promote body homeostasis in conditions as diverse as recovering injured nerve tissue, restraining tumor progression or preventing HIV-1 production (72, 74, 75).

### Modulation of Macrophage Migration by Hormones and Neurotransmitters

The movement of cells is central for proper physiology. During macrophage ontogeny in adults, progenitor cells leave the bone marrow-associated hematopoietic stem cell niche and reach the bloodstream, where they can travel to distant parts of the body. Upon any insult, circulating monocytes are recruited to different tissues by specific chemotactic factors, such as CC- and CX_3_C-chemokine ligands. The receptors of these chemotactic factors also contribute to defining specific monocyte subsets. In humans, classical monocytes are defined by high expression levels of the LPS co-receptor CD14 and the absence of the Fcγ receptor CD16, while expressing high levels of the CC-chemokine receptor 2 (CCR2) and low levels of the CX_3_C-chemokine receptor 1 (CX_3_CR1). In turn, CD16^+^ monocytes can be further divided into two groups, both expressing high levels of CX_3_CR1 and low levels of CCR2. Non-classical cells are CD14^lo^CD16^hi^, whereas intermediate cells are CD14^hi^CD16^lo^ (76). In this regard, several studies have reported that both hormones and neurotransmitters can modulate monocyte or macrophage migration by tuning the levels of target tissue-derived chemokines or the expression of their corresponding receptors (77–79). This lends an additional level of complexity to the control of macrophage behavior in health and disease.

One of these mechanisms includes the α7 and α9-nicotinic acetylcholine receptor (nAChR)-mediated downregulation of CCL2 expression in the brain of the experimental autoimmune encephalomyelitis (EAE) mouse model of multiple sclerosis treated with nicotine. The inhibition of brain-derived CCL2 by nicotine impaired the recruitment of pro-inflammatory CCR2^+^Ly6C^hi^ monocytes during murine EAE—which play a role similar to that of human classical CD14^+^CD16^-^ monocytes—and could be an alternative for mitigating neuroinflammation in clinical settings (80). In addition, the nAChR-dependent modulation of CCL2 expression may also contribute to treating genetic disorders, such as the Duchenne muscular dystrophy (DMD). This dystrophin-related, X-linked condition is worsened by persistent muscle inflammation, including the respiratory muscles, which can cause patient death. A study using *mdx* mice as a model of DMD found that the deficiency of CCR2 lowered the number of muscle-infiltrating inflammatory monocytes and macrophages, therefore, ameliorating disease severity and improving muscle strength (81). Together, this evidence suggests that modulating the migration of monocytes and macrophages through neuroendocrine components may be a valuable tool to control inflammatory diseases.

## Stress-Associated Neuroendocrine Mediators Regulate Macrophage Physiology

A definition of stress can be troublesome, but usually involves an uncontrolled response of the body to aversive changes that may lead to anxiety, emotional tension, or fear. At the cellular level; however, stress is the result of ACTH release by the pituitary followed by the discharge of glucocorticoids produced by the adrenal cortex, along with the release of norepinephrine and epinephrine by the sympathetic-adrenomedullary (SAM) axis of the autonomic nervous system and the adrenal medulla, respectively (82). Glucocorticoids bind their nuclear GR through a mechanism dependent on GR-interacting protein-1 (GRIP1) phosphorylation by cyclin-dependent kinase-9 (83). Activation of this pathway leads to a broad repression of inflammatory-associated genes regulated by the transcription factors NF-κB and AP1, and an upregulation of anti-inflammatory genes, such as the NF-κB repressor glucocorticoid-induced leucine zipper (*Gilz*) (61–63, 84). In turn, signaling by norepinephrine and epinephrine relies on their interaction with β2-adrenergic receptors (β2-ARs), which are seven-pass transmembrane receptors of the family of G-protein coupled receptors. Upon ligand binding-triggered conformational changes, they couple heterotrimeric G_s_ proteins that relay signals to adenylyl cyclase for the production of cyclic AMP (cAMP) and subsequent activation of protein kinase A that may translocate to the nucleus and activate cAMP response element-binding protein to ultimately alter the transcription of target genes (85, 86). When any imbalance disrupting such neuroimmunoendocrine communication occurs, individuals are prone to immunosuppression and increased susceptibility to disease. This is a common occurrence nowadays due to the high number of people living under stressful conditions (87).

It is well established that stress-related mediators vastly affect monocytes and tissue-resident macrophages. For instance, the continued administration of glucocorticoids increases the number of monocytes in the periphery and within the bone marrow, while inducing a substantial reduction of the lymphoid population (88, 89). In culture, however, glucocorticoids were previously known to suppress macrophage growth (90). This apparent discrepancy is found in other monocyte/macrophage processes, such as monocyte trafficking. In fact, Rinehart et al. (91) observed that hydrocortisone succinate impaired the migration of human monocytes in culture, whereas other studies showed that glucocorticoids might stimulate monocyte migration by transiently increasing CCR2 expression in response to moderate physical exercise or transient stress (92, 93). These findings and many others support the notion that drastic changes in glucocorticoid pharmacological activity may occur as their levels and time of exposure increase. Thus, a brief and low-level exposure to glucocorticoids seems to prepare the tissue environment for a greater inflammatory cell response in case of a subsequent insult, whereas sustained high levels of glucocorticoids produce their well known anti-inflammatory properties (Figure 3) (93).

Stressors such as prolonged restraining, cold or heat exposure, footshocks, opioid administration, or psychological challenges disturb macrophage phagocytosis in mice (94–97). In general, these conditions activate both the HPA and SAM axes, while diminishing the levels of pro-inflammatory cytokines (98, 99). For example, cold-induced stress impaired the engulfment of apoptotic thymocytes by LPS-activated macrophages through a glucocorticoid-dependent mechanism that was accompanied by an increase in IL-10 levels. By contrast, treatment of INF-γ-activated macrophages [M(IFNγ)] with glucocorticoids enhanced Fc-mediated phagocytosis of sheep red blood cells in culture (100), thus revealing that the cellular context plays a critical role in macrophage responses. A rise of epinephrine and norepinephrine plasma levels also followed the acute cold stress, but these catecholamines seemed to have no influence on phagocytosis (96). Yet, these findings are at odds with other observations. In particular, many studies have shown that treatments with corticosterone or the glucocorticoid analogs methylprednisolone, dexamethasone, or hydrocortisone augmented macrophage phagocytosis (59, 101), an outcome that was not observed for the mineralocorticoid aldosterone or the sex steroids estradiol or progesterone (102). Taken together, the available information points out that the local or systemic release of stress hormones modulates macrophage ability to phagocytose and may exert a significant impact on both innate and adaptive responses, since the engulfment of apoptotic cells leads to the upregulation of anti-inflammatory genes and cytokines by macrophages (103–105).

Upon phagocytosis, macrophages process and present antigens through MHC molecules to T cells, which might differentiate into unique subsets, such as Th1, Th2, and Th17, each of them bearing specific functions. Stressful conditions may affect macrophage antigen presentation and modify the Th1/Th2/Th17 balance by altering the macrophage cytokine profile and thereby increasing susceptibility to infections or allergic processes (2). For instance, a 4-day exposure of mice to cold water lowered the IFN-γ-induced expression of MHC class II molecules in macrophages (106). Similar findings were observed in restrained mice, in which macrophages showed reduced levels of MHC class II and upregulated concentrations of plasma corticosteroids (107, 108). A rise of serum glucocorticoids and a concomitant decrease in the production of NO through the activation of the HPA axis upon acute cold-induced stress result in the development of an immunosuppressive response that is enhanced by norepinephrine-producing fat-resident macrophages (109–111). Likewise, a Th2-immune response could be observed in heat-stressed mice, which exhibited high plasma levels of norepinephrine and increased macrophage production of CCL2, whose synthesis was controlled by norepinephrine depletion (112). Glucocorticoids, norepinephrine, and epinephrine were able to favor a Th2 profile because they inhibited macrophage synthesis of IL-12, a major Th1-inducing molecule (65, 113). Similarly, corticosteroid-treated monocytes lose their capacity of inducing the production of IFN-γ by CD4^+^ T lymphocytes, whereas stimulating their secretion of IL-4 (114). In addition, stress caused by electric shocks raised plasma corticosterone levels and lowered macrophage antitumor activity, favoring the growth of Ehrlich ascites tumor in a mouse model (95). Other forms of stress induction, such as restraining or corticosterone injection, inhibited the production of TNF-α and reactive nitrogen species by macrophages, which thereby increased susceptibility to *Mycobacterium avium* infection (115).

As part of the HPA axis, there is also an acetylcholine-based suppressive neuroinflammatory-macrophage communication that is centered on the stimulation of the vagus nerve by microorganisms or cytokines (116). Anatomically, these paired nerves gather long-range afferents that convey systemic behavior-changing signals into the brain, and efferents from the medulla oblongata that innervate the heart and numerous visceral organs. In organs such as the liver, pancreas, and the gastrointestinal tract, these nervous fibers can exert relevant control of metabolism and tissue homeostasis (116–118). The release of acetylcholine from synaptic nerve endings targets both muscarinic and nicotinic acetylcholine receptors (mAChR and nAChR, respectively), whose subunits are differentially expressed by monocytes and macrophages depending on species, maturation stage, tissue, and degree of cell activation (119–124). Interaction of the vagus nerve with tissue-resident macrophages is not always direct; in the gut, there is the participation of intervening myenteric neurons scattered around the muscularis layer, whereas the spleen shows no evidence of vagal innervation (125).

Stimulation with acetylcholine inhibits mortality associated with the LPS-induced production of macrophage pro-inflammatory cytokines, such as IL-1β, IL-6, and TNF-α, whereas injuries of the vagus nerve may culminate in uncontrolled inflammatory responses (126). In BALB/c mice, vagotomy led to Kupffer cell-dependent fulminant hepatitis upon intraperitoneal co-injection of LPS and d-galactosamine, an effect that was either reduced by nicotine or exacerbated by α-bungarotoxin, a selective antagonist of the nAChR α7 subunit (127). In agreement with these observations, agonist binding to the nAChR α7 subunit induced the Jak2/STAT3 pathway to suppress resident peritoneal macrophage activation and inflammation (128). Actually, each of these signaling components may have clinical standing to ameliorate the inflammatory output. Notwithstanding, electrical stimulation of the vagus nerve through non-invasive devices is a likely alternative to reduce the numbers of drug-based interventions, as experimentally demonstrated in inflammatory bowel disease, kidney ischemia-reperfusion injury, and rheumatoid arthritis (129–131). Thus, the intricate neuroendocrine regulatory mechanisms of macrophages pose a challenge, but support a role for hormone and neurotransmitter receptors as amenable targets for the development of effective therapeutic strategies based on regulating monocyte and macrophage differentiation, migration, polarization and activation, phagocytosis, or antigen presentation.

## Tissue-Specific Differences in Macrophage Regulation by Stress Mediators

Glucocorticoids effects on macrophages generally influence the resolution phase of inflammation. Interestingly, their actions can be regulated locally by cell-specific particularities, such as receptor availability and metabolism. One of these mechanisms includes the enzymes 11β-hydroxysteroid dehydrogenases type 1 (11β-HSD1) and type 2 (11β-HSD2) that act upstream of the GR receptor and are ultimately responsible for glucocorticoid metabolism. In general, they play opposing roles in a tissue-dependent context. Whereas 11β-HSD1 converts inactive cortisone to active cortisol in the vasculature, adipose tissue, muscle, liver, and brain, the 11β-HSD2 enzyme inactivates cortisol to cortisone in the kidneys and colon. Thereby, glucocorticoid levels can be controlled independently from the systemic axis [reviewed by Chapman et al. (132)].

Macrophages also differentially express these enzymes. In particular, the expression of 11β-HSD2 is low, whereas the levels of 11β-HSD1 are highest in anti-inflammatory macrophages and high in pro-inflammatory cells when compared to resting macrophages (133, 134). This occurs because the cytokines IL-4 or IL-13 are able to upregulate 11β-HSD1 activity, whereas IFN-γ plays a suppressive role. A higher expression of 11β-HSD1 is also found as monocytes differentiate to anti-inflammatory macrophages. Incubation with LPS produces no alteration on the expression of 11β-HSD1 in monocytes, but it can increase enzyme levels in pro-inflammatory macrophages (133). In microglia, LPS does induce an upregulation of 11β-HSD1 expression, but without an apparent change in protein abundance (135). Likewise, the same stressor may also initiate opposing responses in macrophages, which may result in confusion or difficulty of interpretation. For instance, acute cold stress induces a reduction in the phagocytic activity of resting macrophages that is mediated by corticosterone, but an increase in phagocytosis by activated cells that depends on catecholamines (136). On the other hand, chronic cold stress promotes an anti-inflammatory phenotype that correlates with increased expression of 11β-HSD1 (137). In the adipose tissue, these anti-inflammatory macrophages secrete catecholamines to induce thermogenic gene expression in brown adipose tissue and lipolysis in white adipose tissue (111). In turn, neuron-produced norepinephrine activates tissue-protective programs by muscularis macrophages in the intestine, whereas lamina propria macrophages exhibit pro-inflammatory characteristics (138). Those muscularis macrophages respond similarly to norepinephrine as the microglia in the CNS (139, 140).

Together, these observations point out that tissue-specific macrophages have the ability to respond differently to the same neuroendocrine stimulus such as glucocorticoids, and it suggests the existence of an intricate tissue-dependent network of regulation on macrophage function. A pathological condition can, however, modify macrophage response to neuroendocrine mediators. Similar to alveolar macrophages in acute respiratory distress syndrome, synovial macrophages in osteoarthritis or rheumatoid arthritis present high levels of 11β-HSD2, which may contribute to glucocorticoid resistance and the persistence of chronic inflammation (141–143).

## Clinical Relevance of Targeting Macrophages with Neuroendocrine Signals

Both neurons and immune cells are able to sense and respond to exogenous and endogenous challenges, being involved in governing critical homeostatic pathways. It is a remarkable feature the existence of a network allowing these cells to interact with each other *via* cell–cell communication or *via* their main soluble signaling molecules, the neurotransmitters, and cytokines. Considering that these bidirectional communications may be disturbed in immunopathological and neurological diseases, the better understanding of such an intricate body of interactions may help to both unveil new mechanisms of diseases and the search for new therapies. An important homeostatic arm to counteract an inflammatory state is driven by the nervous system *via* triggering the HPA axis and the consequent release of glucocorticoids, and also by activating the sympathetic nervous system to secrete catecholamines. As target cells of these factors, macrophages can be turned to an anti-inflammatory state as glucocorticoids are regarded by their immunosuppressive function and the catecholamines can induce IL-10 macrophage secretion (Figure 3) (65, 144). Additionally, macrophages seem to be regulated by the efferent vagus nerve *via* their nicotinic acetylcholine receptors (145). Therefore, aiming at controlling undesired effects of tissue inflammation, one can envisage that interfering on brain-to-macrophage signaling might be an effective strategy to induce regulatory therapies for inflammatory diseases.

In a second hand, the differentiation program of monocyte/macrophage lineages, and their functional activities, following the differentially acquired polarization status, show a more complex pathway for exploring therapeutically the macrophage to brain signaling. Nonetheless, several studies have demonstrated the critical role of monocyte/macrophage recruitment, activation and polarization in tissue injury and in the outcome of disease progression. Indeed, a change in macrophage function is critical at the distinct phases necessary for the restoration of tissue homeostasis. During the regeneration of skeletal muscle, for example, pro-inflammatory monocyte-derived macrophages induce the proliferation and migration of progenitor myoblasts at the injury site (146, 147). The phagocytosis of dying cells then induces a switch of pro-inflammatory macrophages to an anti-inflammatory phenotype that stimulates myoblast fusion and both the repair of damaged muscle fibers and the formation of new ones (148, 149). The concerted activity of both pro- and anti-inflammatory subsets restores the contractile machinery of muscle fibers and repair the underneath fiber basement membrane. As a proof-of-concept for the delivery of macrophages in clinical application, the coinjection of human myoblasts with pro-inflammatory human macrophages into cryodamaged tibialis anterior muscle of *Rag2*^−/−^γ*C*^−/−^ immunodeficient mice increased muscle cell proliferation and migration, whereas inflammatory cells transited to a resolutive phenotype that supported muscle differentiation through the production of TGF-β after 5 days (146). On the other hand, blocking the anti-inflammatory tumor-promoting activity of tumor-associated macrophages has been reported as an encouraging antitumor therapy (150–152). Interestingly, this can be achieved by the administration of M(LPS + IFN-γ) macrophages in the affected area, which then recruit endogenous macrophages and instruct them into Ly6C^lo^CD11b^hi^F4/80^+^ restorative cells (153).

As for the CNS, one should take into account the broad presence of microglia, the CNS-specific resident macrophages. In this context, as the microglia faces potentially harmful invading entities (e.g., pathogens or tumors), these immunosurveillance cells become activated, triggering a protective inflammatory response. However, dysregulation of this neuroinflammation state might result in tissue damage and neurodegeneration, which show microglial activation as a central pathogenic hallmark (154). This concept points out that approaching microenvironmental polarization in the CNS should be well balanced. Nonetheless, the vast body of fundamental data and clinical studies also indicate that targeting macrophage/microglia activation and polarization should be pursued as a potential therapy for neuroinflammatory diseases. In fact, some clinical and experimental therapeutic approaches for neuroinflammatory conditions are known to induce an anti-inflammatory microenvironment in the CNS with increased expression of type-2 cytokines. Such polarization seems to result in both immunosuppressive and regenerative effects, with the production of anti-inflammatory cytokines and neurotrophic factors, including TGFβ, IL-10, IGF-1, and BDNF (155). Thus, experimental studies aiming at the generation of a Th2 and anti-inflammatory microenvironment by carrying IL-4 expression through viral vectors to the CNS in a murine model of multiple sclerosis, resulted in significant reduction in neuroinflammation and neurodegeneration [reviewed in Ref. (155)]. Similarly, the therapy with synthetic polypeptides that resemble myelin basic protein, known as glatiramer acetate, was reported to induce type-2 cytokines and BDNF production by immune cells, and play an immunomodulatory effect on the relapsing form of multiple sclerosis (156). Accordingly, the presence of anti-inflammatory (or immunosuppressive) macrophages reduces pro-inflammatory components and might offer support for neuronal survival. In fact, enhanced expression of BDNF has been described in activated macrophages and microglia following brain injury (157, 158). Also, a switch to an anti-inflammatory Arg-1^+^CD68^+^ phenotype in microglia and peripherally derived macrophages was shown to correlate with remyelination and to support oligodendrocyte differentiation in a murine model of CNS demyelination (159).

The complex nature of macrophage polarization also shows the potential role of CD14^+^CD16^+^CD163^+^CD204^+^CD206^+^CD209^−^ macrophages in the resolution of an inflammatory state. Anti-inflammatory M(M-CSF) or M(GC) macrophages highly express the MerTk receptor for apoptotic cells (55), a feature that points out this phenotype as a further target in neuroinflammatory conditions as the neuronal protection and survival requires an efficient clearance of apoptotic cells and debris. Engulfment of apoptotic cells by M(M-CSF + IL-10) macrophages integrates resolution of inflammation with proper tissue repair and the consequent waning of the neurodegeneration process (55).

The immunomodulatory role of the cholesterol-lowering drug atorvastatin on macrophage function has also been explored as a potential anti-neuroinflammatory agent. In a murine model of traumatic brain injury, this drug inhibited microglia/macrophage activation and showed enhanced anti-inflammatory polarization (160). Interestingly, atorvastatin has been reported to downmodulate activation of a blood monocyte subset that seems to be involved in HIV-1-associated neurocognitive disorders (161). Since this compound can accumulate in the CNS, its effects on both recruited inflammatory monocytes and microglia have been a matter of clinical investigation (https://clinicaltrials.gov/, ID: NCT01600170). Altogether, these data gather some interesting concepts derived from basic studies regarding the regulation of macrophage activities on homeostatic and pathological conditions. More importantly, the evidence of macrophage interplay with components of the nervous system and their functional role in neuroinflammatory conditions mounts progressively, hopefully enticing new clinical studies on more efficient treatment of cancer, inflammatory and neurodegenerative diseases.

## Author Contributions

AJ conceived the review subject, analyzed transcriptome data, and wrote the paper. VC-d-A and JT wrote the paper. WS conceived the review subject and wrote the paper. DB-H and IR conceived the review subject, coordinated the work, and wrote the paper. Artwork by AJ and IR.

## Conflict of Interest Statement

The authors declare that the research was conducted in the absence of any commercial or financial relationships that could be construed as a potential conflict of interest.

## Supplementary Material

Figure S1 Monocytes and macrophages express many neuroendocrine-related genes. Gene expression of selected neurotransmitters and hormone receptors (columns) in distinct monocyte and macrophage populations (rows) distributed throughout the body. The ImmGen dataset was plotted as heatmap with hierarchical clustering as described by Beyer et al. (162), using a coefficient of variation of 20%. Levels of expression are represented by colors in which red, white, and blue indicate high, intermediate, and low intensities, respectively.
