# Supplementary figures and images for: Neuroendocrine Control of Macrophage Development and Function
Source: Front Immunol. 2018 Jun 25;9:1440. doi: 10.3389/fimmu.2018.01440 (PMC6026652; doi:10.3389/fimmu.2018.01440)

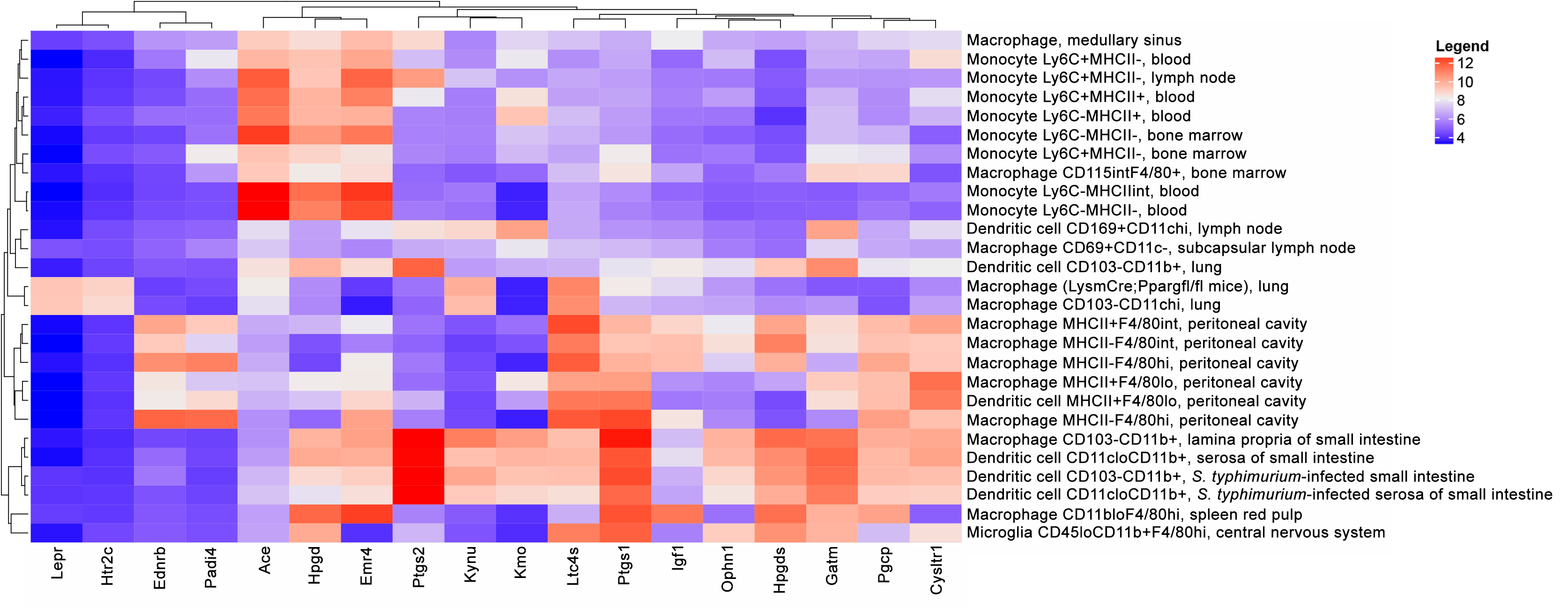

Supplement: Figure S1 — Monocytes and macrophages express many neuroendocrine-related genes. Gene expression of selected neurotransmitters and hormone receptors (columns) in distinct monocyte and macrophage populations (rows) distributed throughout the body. The ImmGen dataset was plotted as heatmap with hierarchical clustering as described by Beyer et al. (162), using a coefficient of variation of 20%. Levels of expression are represented by colors in which red, white, and blue indicate high, intermediate, and low intensities, respectively. [file Image_1.TIF]
